# Does premature birth affect Brazilian parents’ practices related to infant positioning?

**DOI:** 10.1590/1984-0462/2024/42/2022163

**Published:** 2023-07-24

**Authors:** Anilsa Suraia Pedro Gaspar Francisco, Maylli Daiani Graciosa, Sheila Cristina da Silva Pacheco, Anelise Sonza, Luciana Sayuri Sanada

**Affiliations:** I Universidade do Estado de Santa Catarina, Florianópolis, SC, Brazil.

**Keywords:** Parent guidance, Motor development, Infant, sleeping position, Body position, Mother-infant interaction, Orientação parental, Desenvolvimento motor, Posicionamento infantil dormindo, Posição corporal, Interação mãe-lactente

## Abstract

**Objective:**

To verify whether the time spent in prone, supine, or seated positions differed between term and preterm infants; and to determine whether a single verbal guidance session for parents changed the time spent in different positions, and, consequently, the motor development scores, after one month in preterm infants.

**Methods:**

Sixty-one infants from a full-term and preterm group from Brazil were included. Motor development was assessed by the Alberta Infant Motor Scale (AIMS) and the parents registered the time spent in each position on a 24-hour schedule. A month after verbal guidance, a second assessment was performed only on the preterm infants.

**Results:**

The positioning times awake determined for the full-term and preterm parents were similar. Preterm infants spent more time in the prone sleeping position (2.1 *vs.* 0.8 h; p=0.037) than full-term infants. The AIMS percentile scores did not differ significantly between the groups. For preterm infants, the time spent in all positions did not change during the second assessment (n=18).

**Conclusions:**

The fact that some parents position their infants in the prone posture during sleeping periods reinforce the importance of parental education approaches for sudden infant death syndrome (SIDS) prevention during the first months of life. The verbal guidance provided to parents of preterm infants did not influence the AIMS percentile and time spent in various positions but increased preterm parents’ confidence in placing their infants in a prone position to play.

## RESUMO

**Objetivo:**

Verificar se o tempo nas posições prono, supino ou sentado difere entre lactentes a termo e pré-termo; bem como determinar se uma única sessão de orientação verbal aos pais alterou o tempo despendido nas diferentes posições e, consequentemente, o escore de desenvolvimento motor, após um mês em lactentes pré-termo.

**Métodos:**

Foram incluídos 61 lactentes brasileiros nos grupos a termo e pré-termo. O desenvolvimento motor foi avaliado pela Alberta Infant Motor Scale (AIMS), e os pais registraram o tempo despendido em cada posição em uma linha do tempo de 24 horas. Um mês após a orientação verbal, uma segunda avaliação foi realizada apenas nos lactentes pré-termo.

**Resultados:**

O tempo de posicionamento acordado determinado pelos pais de lactentes a termo e pré-termo foi semelhante. Os lactentes pré-termo passaram mais tempo dormindo na posição prona (2,2 *vs*. 0,8 h; p=0,037) do que os lactentes a termo. Os escores de percentil AIMS não diferiram significativamente entre os grupos. Para lactentes pré-termo, o tempo despendido em todas as posições não se modificou durante a segunda avaliação (n=18).

**Conclusões:**

O fato de alguns pais posicionarem os lactentes em decúbito ventral durante os períodos de sono reforça a importância das abordagens educativas parentais para a prevenção da síndrome da morte súbita do lactente (SMSL) durante os primeiros meses de vida. A orientação verbal fornecida aos pais de prematuros não influenciou o percentil da AIMS e o tempo de permanência em várias posições, mas aumentou a confiança dos pais de lactentes prematuros em utilizar a posição prona para brincar.

## INTRODUCTION

Preterm birth is a biological factor that not only increases the risk of motor development (MD) delay but may lead to atypical mother-infant interactions.^1^ Mothers of preterm infants usually report insecurity in interacting with their children in the first months of age, especially after hospital discharge.^2^ Since mother-infant interactions influence motor skill acquisition,^3^ the need for family-centered parent education programs to enhance parents’ abilities and self-confidence in caring for preterm infants has been identified by several studies.^2,4,5^

A Brazilian study suggested that educational programs for MD in premature infants should inform parents about the importance of body position change throughout the day, increasing the time spent in the prone, sitting, and standing positions, and reducing the time spent in the supine posture.^4^ “Tummy time” (TT) recommendations are associated with the greater acquisition of motor skills in infants,^6^ reduction of body mass index, prevention of cranial asymmetry and global development.^7^ The TT campaign is a consequence of the “Back to Sleep” program outcomes, whereas the warning to avoid prone posture for sleep made caregivers avoid it even when the infant is awake.^8^ In Brazil, guidelines for this campaign have been disseminated since 2009, and even though the document released by the Brazilian Society of Pediatrics on sudden infant death syndrome (SIDS) in 2018 describes wakeful prone practice under supervision, the lack of standardization in the recommendations on the duration and frequency of this practice is notable.^9^

Furthermore, Koren et al.^10^ found that, in addition to SIDS-related fears, confusion regarding the guidelines is among the main barriers to parents’ adherence to TT. Thus, investigation of parental practices related to infant positioning in the Brazilian population is essential. Regarding the particular mother-infant interactions in the context of premature birth, Valentini et al.^4^ highlighted the influence of the gap in studies addressing preterm infants’ daily routines in the limited understanding of the relationship between parental practices and MD. Thus, intermediation of parents’ practices to promote MD would first require an understanding of their choices related to infant positioning throughout the day.

These findings can help clinicians to develop guidelines for preterm infant positioning in family educational programs. This study aimed to verify whether the time spent in prone, supine, or seated positions differed between term and preterm infants; and to determine whether a single verbal guidance session for parents changed the time spent in different positions, and, consequently, the MD scores after one month in preterm infants.

## METHOD

The intentional and non-probabilistic sample consisted of infants from Florianópolis/SC, Brazil, with corrected or chronological ages of four months (±7 days) who were allocated into two groups. The preterm group (PTG) (n=31) included infants with a gestational age (GA) <37 weeks. The full-term group (FTG) (n=31) included infants with a GA ≥37 weeks, five-minute MD Apgar score higher than 7, and birth weight higher than 2.5 kg. Infants with physical, cardiological, neurological disabilities; genetic syndromes; or who were not fully evaluated by the Alberta Infant Motor Scale (AIMS) were excluded from the study. The study was approved by the Ethics Committee on Human Research (protocol number: 432.136).

A self-assessment questionnaire was administered to collect infants’ and mothers’ data, gestational aspects, and information about infant positioning. The infant-positioning section included items that asked parents if they had any concerns about some position, and if they had ever received professional instructions about this practice. In addition, a 24-hour schedule developed by Graciosa et al.^11^ was included to understand how long the infants remained in each position during the day. To complete this schedule, parents were required to remember the infant’s most frequent position during the previous week. This schedule was divided into 24 one-hour periods that were filled with one position option: asleep in prone, supine, right, and left lateral decubitus; awake in prone, supine, right, and left lateral decubitus; and sitting with and without support.

The AIMS assessed the gross MD of the infants. This observational scale can be applied to full-term and preterm infants, and it contains 58 items that are divided into prone (21 items), supine (9 items), sitting (12 items), and standing (16 items) subscales.^12^

The data collection of the present study occurred for a 12 months period. A comparative case-control study was designed to understand whether parents of preterm infants practice different body-positioning for their infants than those of full-term infants. In addition, a quasi-experimental pretest-posttest design was adopted to investigate the effect of verbal guidance from a healthcare professional to parents on daytime positioning in the PTG. Data collection consisted of two assessments on a physical therapy clinic. The first one was conducted in both groups at four months of chronological or corrected age (±7 days), and the second one was conducted only for infants from PTG at the fifth month (±7 days) of corrected age.

Initially, parents received instructions about how to fill in the questionnaire; for filling the 24-hour schedule, at this point, the parents received positioning options in usual terms (belly down or up, right side or left side, sitting with support, or without support). Then AIMS assessed infants’ gross MD.

Subsequently, on the basis of the AIMS percentile result, parents received verbal guidance. The theoretical basis for this guidance was the perspective of complex dynamic systems that considers variability crucial for motor skills’ emergence and adaptation processes.^13^ Thus, parental guidance emphasized postural changes, since the variable experiences of body orientation in different positions produce the emergence of specific posture control and motor behaviours. First, parents received explanations about the results of the AIMS assessment and about future skills that the infant could develop in the different postures. Second, the researcher highlighted the importance of parents in placing the infants in different positions while awake during the day. Furthermore, parents were instructed to play with their infants on the floor, especially in the prone position for at least 30 min per day with parental interaction and supervision. Parental interaction as showing toys, talking, singing, and interacting with facial expressions were recommended as it increases connection and socio-emotional development.^5^ Third, the examiner explained the different places and surfaces where the infant could stay and their advantages and disadvantages (bed, stroller, bouncer, infant car seat, activity gym, rugs, and playmats). Parents were instructed to leave infants on safe, spacious, and firm surfaces and to provide toys during postural practice that would allow for reaching, changing positions, and sensory stimulation.

To verify the effects of this verbal guidance on parental preterm infant-positioning practices, and MD, a second assessment was performed a month later. The second assessment consisted of the same procedures as the first (parents’ self-assessment questionnaire, AIMS assessment, and new MD guidance).

Data were analysed using the Statistical Package for the Social Sciences version 20.0 (IBM SPSS^®^, USA) with a significance level of 5%. Comparison between groups was performed using Student’s t-test for independent samples and the Mann-Whitney U test.

Student’s t-test for paired samples and Wilcoxon test were used to verify differences in infants’ positioning time and AIMS scores between the first and second assessments in the PTG. Spearman’s correlation coefficient was used to test for the presence of a linear association between the differences in prone positioning time after verbal guidance and the corresponding differences in the AIMS percentile.

## RESULTS

Table 1 shows the infants’ characteristics and Table 2 presents the data for the time spent awake in different positions, and AIMS scores for both groups. The PTG spent longer periods sleeping in the prone position than the FTG (p<0.05). The FTG spent more time sleeping in the supine position (p<0.01).

**Table 1.** Characteristics of preterm (n=31) and full-term (n=31) infants.

Characteristics	Preterm		Full-term		
	n	(%)	n	(%)	
Female/Male (n)	16/15	51.6/48.3	16/15	51.6/48.3	
Breastfeeding infants (n)	21	67.7	28	90.3	
	**Mean± SD** **Or median [IQ]**	**CI95%** **Or min-max**	**Mean± SD** **Or median [IQ]**	**CI95%** **Or min-max**	**p-value**
Gestational age (weeks)*	33 [2]	27–36	39 [2]	37–42	0.001^‡^
Chronological age (months)*	5.7 [0.5]	5.00–7.25	4.0 [0.5]	3.75–4.25	0.001^‡^
Birth weight (kg)^†^	1.6±0.5	1.49–1.88	3.1±0.5	3.01–3.38	0.001^‡^
Weight at 1^st^ assessment date(kg)*	6.2 [1.3]	3.90–8.40	6.4 [0.9]	2.60–7.50	0.272
1^st^ minute Apgar score*	8.0 [1]	3–9	9 [1]	4–10	0.001^‡^
5^th^ minute Apgar score*	8.0 [1]	5–-9	9 [1]	7–10	0.001^‡^

CI: confidence interval; SD: standard deviation; *Mann-Whitney U test; ^†^student’s t-test for independent samples; ^‡^significant difference.

**Table 2.** Comparison of spent time awake and asleep in different positions, and Alberta Infant Motor Scale (AIMS) scores between preterm (n=31) and full-term infants (n=31).

	Preterm		Full-term		p-value
	Mean± SD or median [IQ]	CI95% or min-max	Mean± SD or median [IQ]	CI95% or min-max	
Asleep time in prone (hours)*	0 [6]	0–11	0 [0]	0–12	0.037^‡^
Awake time in prone (hours)*	2 [2]	0–8	1 [2]	0–6	0.073
Asleep time in supine (hours)*	12 [ 5]	2–16	14 [ 3]	0–21	0.003^‡^
Awake time in supine (hours)^†^	5.5±2.9	4.49–6.60	4.5±2.9	3.5–5.6	0.190
Seated time (hours)*	3 [3]	0–10	4 [4]	0–9	0.815
AIMS total score^†^	16.0±3.0	14.97–17.16	15.4±3.1	14.3–16.6	0.432
AIMS percentile (%)*	70 [40]	7–90	55 [44]	15–90	0.065

CI: confidence interval; SD: standard deviation. *Mann-Whitney U test; ^†^student’s t-test for independent samples; ^‡^significant difference.

The average time mothers spent with their infants was 21.6 (±5.14) hours per day. All parents affirmed they had not received previous guidance regarding the infants’ MD and body positioning.

For the second assessment, all infants of the PTG were invited; however, only 18 returned. Table 3 shows the characteristics of those who participated in both assessments. In the first assessment, seven parents (38.9%) reported fears related to positioning their infants. Six parents were concerned about positioning the infants in the prone position, and one parent, in the supine position. In the second assessment, two parents (11.1%) still demonstrated concerns regarding the prone position. In the first assessment, 15 (83.3%) infants were classified as having typical MD, and three (16.7%) infants had atypical MD. In the second assessment, 16 (88.9%) infants were classified as having typical MD, and two (11.9%) infants still had atypical MD.

**Table 3.** Characteristics of the preterm infants (n=18).

	Result	
Female/male (n)	10/8	
Gestational age (weeks)	31.5±2.4	
Birth weight (kg)	1.6±0.6	
1^st^ and 5^th^ minutes Apgar score	7.3±1.7/8.2±1.0	
	**1^st^assessment**	**2^nd^ assessment**
Weight at assessment dates (kg)	6.3±1.2	7.0±1.2
Chronological age (months)	5.9±0.6	7.1±0.6
Corrected age (months)	3.9±0.2	5.1±0.2

Values expressed in mean +/- standard deviation.

**Figure 1.**
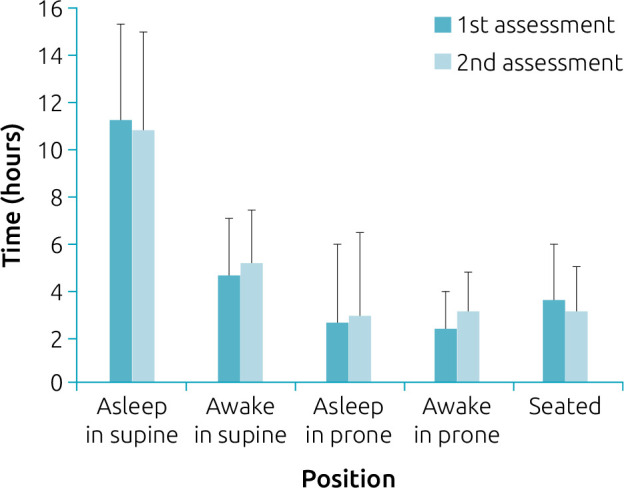
Preterm infants’ time spent in prone, supine, and seated positions within a 24-hour period before and after motor development verbal guidance (n=18).

The infants’ time spent in prone, supine, and seated positions over 24 hours in both assessments are shown in Figure 1. The supine position was the preferred position during the asleep and awake periods in both assessments. The infants spent less time in the prone position in the first assessment. In the second assessment, they spent similar time in the prone and seated positions when awake. There was no significant difference in the time spent in the prone, supine, or seated postures between assessments (p>0.05).

Table 4 presents a comparison of the AIMS scores between the assessments. Significant differences were observed for all AIMS subitems, with higher scores on the second assessment. The AIMS percentile did not show a significant difference between the assessments, and no linear correlation was observed between the difference in the time spent in the prone position after verbal guidance and the corresponding difference in the AIMS percentile (rho=0.29, p=0.241).

**Table 4.** Comparison of the Alberta Infant Motor Scale scores before and after verbal guidance assessments for preterm infants (n=18).

AIMS scores	Assessment		p-value
	Before VG	After VG	
Prone	5.3±1.9	8.2±1.8	<0.001*^,†^
Supine	5.2±1.3	6.9±1.4	<0.001*^,†^
Sitting	3 [1]	4 [2]	<0.001*^,‡^
Standing	2 [1]	3 [1]	0.014*^,‡^
Total score	15.5±3.1	22.1±4.2	<0.001*^,†^
Percentile	32.4±20.5	41.3±27.2	0.078^†^

AIMS: Alberta Infant Motor Scale; VG: verbal guidance; *significant difference; ^†^student’s t-test for paired sample; ^‡^Wilcoxon test.

## DISCUSSION

To the best of our knowledge, this study is the first to show the differences between Brazilian preterm infants’ time spent in the prone, supine, or seated postures within a 24-hour daily routine compared to full-term infants. The results showed a difference between the groups only during the sleep periods. Contrary to our expectations, the average time that the PTG slept in the prone position was longer than the FTG. Similar to our results, Hwang et al.^14^ found that late preterm infants were less likely to be placed in the supine position for sleep than full-term infants. Moreover, Pretti et al.^15^ observed that, while 11.1% of preterm infants slept in prone position, none of the infants in the FTG slept in this position. One of the reasons for this result could be that parents of preterm infants already had the opportunity to observe their infants experimenting with a variety of postures in neonatal intensive care units, especially in prone position, which is frequently adopted as a strategy to manage stress and improve respiration in infants.^16^

The fact that some infants in both groups were positioned prone during sleep deserves further attention. Prone sleeping position must be avoided to reduce the risk of SIDS until one year of age;^17^ furthermore, preterm birth is associated with a higher risk of SIDS.^17,18^ Thus, our results demonstrate the need for parental education regarding this syndrome.

For both groups, the shortest time spent during the awake period was during the awake period was in the prone position. Similarly, Hesketh et al.^19^ found that the majority (70%) of the studied 4-month-old infants did not achieve the recommended amount of TT for a day. Even though the average time spent in prone positioning for both groups in the present study exceeded 30 min, which is the minimum recommended,^20^ the mean value may have been influenced by the values of infants that possibly remained in this posture for longer periods of time.

Despite the fact that caregivers’ knowledge is not associated with prone positioning practice, the lack of standardization on published educational materials on TT can limit its implementation.^21^ Indeed, caregivers feel enhanced self-efficacy to place the infant in prone posture for play when they understand the goals and benefits of TT practice for infants.^22^ Additionally, researchers identified some barriers for parents to adhere to TT recommendations, including negative infant effects, scheduling trouble regarding time, and lack of self-efficacy to the practice.^22^ Therefore, educational programs focused on promoting TT practice in addition to providing parents technical knowledge on the prone position must include guidance on practical aspects as much as interaction with the infant, time and space organization.

The PTG and FTG did not show any differences in AIMS scores. Accordingly, since the time spent in the prone, supine, and seated postures was similar in both groups, we did not expect differences in motor acquisition between the PTG and FTG included in this study. In a cross-sectional study, Valentini et al.^4^ found differences in the AIMS subscale scores between full-term and preterm groups. Particularly for groups of infants with the same age as the present sample, i.e., four months, they did not find statistically significant differences for the prone, supine and standing AIMS subscale scores. Importantly, preterm infants in both studies showed moderate or late prematurity, and impairments in MD are more common in infants with extreme prematurity.^23,24^

The present study also aimed to verify whether a single verbal guidance session for parents about MD could influence infants’ positioning time and AIMS scores. The results showed that the time preterm infants spent in each position did not significantly change after providing guidance to parents. Consequently, the MD percentile did not significantly change between the two assessments.

Even though not statistically significant, a difference was noted in body positioning between the two assessments. The awake time spent in prone and supine positions increased, while that in sitting position decreased. The verbal guidance encouraged prone positioning with safety during the wakeful period, in agreement with Palmer et al.^25^ Their results demonstrated that a single meeting of caregivers with healthcare professionals for specific recommendations on playing in the prone position can yield greater adherence by parents and the full-term infants’ acceptance of this posture.^25^ In our study, the number of parents with fear of positioning their infants in the prone posture dropped from 33.3% in the first assessment to 11.1% in the second one. Thus, our results suggest that offering verbal guidance to parents can be a strategy to increase their confidence to increase prone positioning of the PTG.

Previous studies have shown that infants within the age of three, four, and six months spent longer times seated or in supine position than in prone position while awake.^26,27^ The present study found the same result in the first assessment, but, in the second assessment, the results demonstrated a different distribution of time spent in these positions. One month after the verbal guidance, even though the time spent in the supine position was longer, the wakeful time spent in the prone and seated postures was similar. Therefore, the instructions provided to parents in the present study addressed the recommendation from Valentini et al.^4^ in terms of promoting variability in postural practices, increasing the balance in the length of stay in different positions in the second assessment. Since MD changes depend on the variability of movement patterns,^4^ infants should experience different positions. Thus, it is of importance to educate infants’ parents about MD and its positive relationship with postural variations at awake periods during the day.

When comparing the findings obtained before and after verbal guidance, a significant increase was observed in the AIMS subscale and total score, which was expected due to the continuous rise with age in motor skill acquisition in the PTG.^28^ Notably, the AIMS’ prone subscale score increased the most in comparison with the other positions, probably due to the increased time spent in the prone position during the month before the second assessment. Corroborating these results, Valentini et al.^4^ found that motor acquisition in the prone position for preterm infants showed a significant increase, especially from four to eight months of age.

The AIMS percentile showed no significant changes between the two assessments, which means that verbal guidance did not interfere in MD of the PTG included in this study. However, the percentile value increased between the two assessments. The short time interval between the two assessments in the present study may be one reason why there was no significant difference in the AIMS percentile, given that other studies that investigated the influence of MD interventions in infant population adopted greater intervals.^28,29,30^

Further, the present intervention intended to advise parents only through verbal guidance, and did not use any kind of paper-based material or audio-visual sources. In the specific case of TT guidance, for example, many caregivers suggested that a take-home manual of wakeful prone positions with images would be helpful for them to remember how to practice variations at home.^23^ Therefore, forthcoming studies can consider focusing on elaborations of infant-positioning guidance approaches with different strategies in addition to verbal advice.

The small sample size because of the loss on the quasi-experimental study represents a limitation, as does the absence of a control group. The high drop-out rate (41%) between the first and second evaluation is also a limitation of this investigation which decreased the representativeness of the present sample. Because the preterm infants in this study were only classified as moderate or delayed, we suggest that future studies should also investigate the findings for extremely premature infants. Since the majority of infants included in this study had normal MD, it would be worthwhile to assess the effects of this intervention on the practices of parents of infants with motor delay. Other important limitation is the 24-hour schedule developed by Graciosa et al.^11^ to evaluate how long the infants remained in each position during the day, which may lead to memory bias.

Specific body-positioning times determined by full-term and preterm parents are similar. The fact that some parents position their infants in the prone posture during sleeping periods reinforces the importance of parental education approaches for SIDS prevention. Gross MD was similar in preterm and full-term infants. The verbal guidance provided to parents of preterm infants did not influence the AIMS percentile and time spent in various positions but increased preterm parents’ confidence in placing their infants in a prone position to play.

## Data Availability

The database that originated the article is available with the corresponding author.
